# T14. ASSESSING DIFFERENCES IN INFLAMMATORY MARKERS BETWEEN FIRST EPISODE PSYCHOSIS PATIENTS AND HEALTHY CONTROLS: THE IMPORTANCE OF CONTROLLING FOR CONFOUNDING FACTORS

**DOI:** 10.1093/schbul/sby016.290

**Published:** 2018-04-01

**Authors:** Annalisa Giordano, Valeria Mondelli, Mitul A Mehta, Steve Williams, Carmine M Pariante, Paola Dazzan

**Affiliations:** Institute of Psychiatry, Psychology & Neuroscience, King’s College London

## Abstract

**Background:**

Although there is a cumulative evidence for increased level of markers of inflammation in psychosis, it has not been conclusively proven yet whether this is due to the disorder in itself or to other factors. One reason for this is the lack of studies that have controlled for major confounding factors such as obesity, smoking, antipsychotic use and stress. The BMI, as an indirect measure of body fat level, and tobacco smoking are known to play a role in modulating the immune system, but little research has been done in this area in patients with psychosis. The aim of this study was to investigate the differences in markers of inflammation and neuroplasticity between FEP patients and HC while controlling for a priori confounding factors, such as BMI and tobacco smoking.

**Methods:**

Nineteen First Episode Psychosis (FEP) patients and 21 healthy controls (HC) matched for age, gender, ethnicity and marital status were recruited in South London (UK). Blood samples were collected to measure High Sensitivity C-Reactive Protein (hs-CRP), Interleukin (IL)-6, IL-8 and Brain-Derived Neurotrophic Factor (BDNF). Body Mass Index (BMI) was also assessed. Moreover, patients were asked whether they were tobacco smokers. Differences in continuous variables were analysed using independent samples t-tests. Categorical variables were assessed using the chi-square (χ2) test. One-way ANCOVAs were conducted to assess difference between FEP patients and HC in markers of inflammation and BDNF while controlling for the effect of BMI and tobacco smoking. All analyses were conducted using IBM SPSS statistical software version 23. The significance value for all tests was set at α = 0.05.

**Results:**

FEP patients had higher serum level of hs-CRP compared to controls (N = 19, M = 2.29 mg/L, SD = 2.76; N = 21, M = 0.56 mg/L, SD = 0.41; respectively, t(27.79)=-2.41, p=0.02), suggestive of a hyperactivation of the immune system. There was no significant difference in IL-6, IL-8 and BDNF serum levels between the two groups. BMI was significantly higher in patients than controls (N = 19, M = 27.67, SD = 4.91; N = 21, M = 23.55, SD = 3.31; respectively, t(38)=-3.14, p=0.003) and there was a significantly higher number of smokers in the FEP patient group than in the HC group (smokers= 57.9% of FEP patients; smokers=14.3% of HC; χ2(1)=8.34, p=0.004). The one-way ANCOVAs showed that there was not significant effect of group on hs-CRP, IL-6, IL-8 and BDNF values when controlling for BMI and tobacco smoking. In fact, BMI was significantly related to the hs-CRP level, F(1,36) = 7.20, p = 0.01.

**Discussion:**

We found that BMI and tobacco smoking may be important confounding factors when investigating disease-related alterations in the levels of hs-CRP, IL-6, IL-8 and BDNF. More studies are needed to assess the role of potential confounding factors on markers of inflammation in psychosis and to understand the role of immune activation in the pathophysiology of these disorders.

